# Efficient Heritable Gene Expression Readily Evolves in RNA Pools

**DOI:** 10.1007/s00239-017-9800-1

**Published:** 2017-07-01

**Authors:** Michael Yarus

**Affiliations:** Department of Molecular, Cellular and Developmental Biology, University of Colorado, Boulder, CO 80309-0347 USA

**Keywords:** Chance utility, Constant hazard, Cross-templating, Origin of life, RNA catalysis

## Abstract

Heritable gene expression arises readily in a simple non-genetic system employing known small-RNA biochemistry. Pooled cross-templating ribonucleotides show varied chemical competence on which selection acts, even calculating only minimal effects. Evolution can be quick—computed progress toward encoded gene expression can require only days or weeks for two millimolar, partly activated complementary 5′ ribonucleotides. After only one product selection cycle, early templating can become prevailing pool behavior. Subsequently, a selected templated product is efficiently amplified as a pool ages, frequently accumulated in the same order of concentration as incoming nucleotides. Pools spontaneously favor templating because sporadic nucleotide accumulations increase it—and selection increases templating in pools of all ages. Nonetheless, templated chemical competence appears most easily in young pools. Pool history is critical—pools can perish from periodic hazards (like tides), or alternatively, from hazards roughly constant in time (like rainfall). Selection is greatly enhanced in constant hazard pools—more effective if pools have varied ages. Stronger selection is disproportionately more effective. Selected evolutionary change has an uncomplicated molecular basis—progress from chemical product synthesis to templated, proto-genetic inheritance exploits identity between templating and entropic catalysis. Though discovered by computation, selection of an elevated product of template catalysis is plausible, independent of any chemical or mathematical assumption. Selected chemical variation before genetics (chance utility) therefore inaugurates inheritance, even when hindered by unstable, dilute nucleotides, erratically supplied in undependable quantities. Remarkably, such uncontrolled conditions are not necessarily hostile, but can instead accelerate appearance of primordial gene-like behavior.

**Electronic supplementary material:**

The online version of this article (doi:10.1007/s00239-017-9800-1) contains supplementary material, which is available to authorized users.

## Introduction

### The Origin of Life

The origin of life on Earth is more appropriately a succession of molecular innovations, rather than a single event. Each innovation (reproduction, metabolism, cellularization…) has modern partisans whose disagreements about priority can stem from the fact that every such innovation was a logically indispensable, thus equally important, step toward a complete biological repertoire. However, there is recent progress at the early and late ends of a credible origin chain. Simple biomolecules of different classes, including nucleotides, can be obtained from reactions involving HCN and H_2_S—plausibly primordial and plausibly co-existent in one landscape (Sutherland 2016). In more recent times, protocells might have encapsulated RNA-like replicators that did not require catalysis (Prywes et al. 2016). Here, we imagine a primordial middle epoch when molecules began to exhibit biological properties, in particular, an inheritable phenotype.

### A First Genetic System

Biological expression and inheritance is verified below by calculation, using only already-known activities of ribonucleotides and small RNAs. Because the complexity of larger RNAs is a major barrier to their function in primordial environments (Yarus 2015), simplicity is a major priority.

### A Simple First Gene Product

The first such simplification is that the primordial chemically active “gene product” is related to present-day coenzymes, 5′–5′ dinucleotides, rather than to a larger RNA (Yarus 2011). Because primordial chemical synthesis of nucleobases usually includes varied, possibly chemically reactive derivatives with similar base pairing (Oro 1961; Levy and Miller 1999), the same geochemical sources that yield pA can also yield a reactive pA*. Together such pA and pA* nucleotides comprise the ingredients for a coenzyme-like molecule. Modern coenzymes indeed have independent chemical activities, usually a subset of their reactions as a part of a protein enzyme (Yarus 2011). The model coenzyme synthesis used here is production of AppA and GppG, whose encoded (templated) and chemical (untemplated) synthesis rates are known (Puthenvedu et al. 2015; Majerfeld et al. 2016).

### Cross-Templating RNAs Encode the Simplified Product

**Scheme 1:**
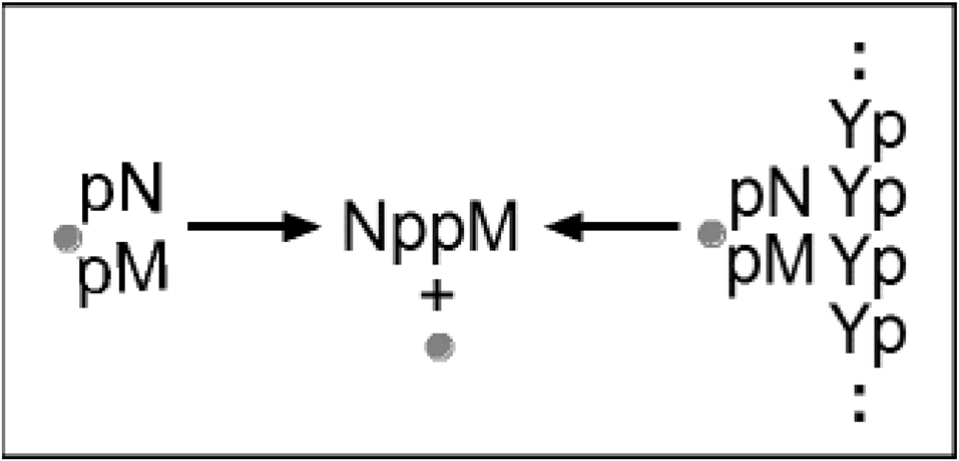
Two routes to NppM. Chemical (chem, Scheme *left*) and templated (temp, Scheme *right*) synthesis of cofactor-like RNAs. pN and pM are related to 5′ purine nucleotides, both complementary to a pyrimidine (pY) polymer. NppM is a 5′–5′ linked ribodimer product, a hypothetical congener to coenzymes like NAD (Yarus 2011). The *circular gray symbol* is a phosphate-activating group, 2me-imidazole in our experiments (Joyce et al. 1984), which can be thought of as a substituent of either 5′ pN or 5′ pM. The two purines stack and the unactivated phosphate readily attacks an adjacent activated one in a helical complex to yield the N^5′^pp^5′^M pyrophosphate (modeled in Puthenvedu et al. 2015)

A gene-like system of reactions with simpler requirements than modern expression is easily envisioned (see Majerfeld et al. 2016). Cross-templating RNAs encode 5′–5′ coenzyme-like ribonucleotide dimers whose nucleobases are complementary to homopolymeric 3′–5′ single-stranded RNA templates. Poly(U) encodes A^5′^pp^5′^A (Puthenvedu et al. 2015) and poly(C) encodes G^5′^pp^5′^G (Majerfeld et al. 2016), using a mixture of activated and normal 5′ purine nucleotides. Co-existing chemical (left, Scheme 1) and templated (right, Scheme 1) pathways yield the coenzyme congener NppM. Synthesis occurs in free, stacked nucleotides (chem or chemical), or in base-paired, stacked nucleotides (temp or templated). Coenzyme-like products might perform metabolic chemistry, thereby supporting a novel phenotype, as modern coenzymes still do (White 1976; Yarus 2011).

Thus, in Scheme 1 homopolymer RNAs are viewed as simplified genes and dimer coenzyme-like molecules as simplified gene products (Yarus 2011). Synthesis rates and stabilities for Scheme 1 reactions come from cross-templating small RNAs (Majerfeld et al. 2016), and estimation of their lifetimes (see “Methods”). A–U (Puthenvedu et al. 2015) and G–C base pairs (Majerfeld et al. 2016) both form synthetically competent cross-templating complexes. Scheme 1 simplifies the chemical events analyzed, but a more explicit chemical scheme and the method for integration of their timed behavior is available (Scheme 3; Supplementary Information).

### A Single-Stranded Homopolymer Gene

The second simplification (right, Scheme 1) is that a gene-like template can be as simple as a single, monotonous sequence, strand of RNA. Beneficial reactions of such primordial “genes” would be available to any system which retains them—without independent, complementary RNA replication. There is no specific molecular rationale for simultaneous evolution of replication and expression, because they require different molecular events. Given that idea, the simpler process will likely appear first. Complementary replication seems more complex than cross-templating, and will probably be the later event, after expression. Indeed, there seems little selection for replication at all without a preexisting expression mechanism to make replication advantageous.

### A Mineral-Aided Origin for Rudimentary Genes

The RNA template is the most complex reactant in Scheme 1, but there is a ready geosynthesis for it. Such simple polymers arise by exploiting preexisting order in the interior of clay minerals (Ferris and Ertem 1992), which helps to lengthen chemically synthesized linear RNAs (Ferris et al. 1996) made from activated 5′ nucleotides. In this way, earliest gene action might rely on chance acquisition of environmental single-stranded RNAs that subsequently encode chemically reactive, coenzyme-like gene products (Scheme 1; Yarus 2011; Majerfeld et al. 2016). This sequence also allows time for a later, complex complementary replication mechanism to co-evolve with persistent gene function.

### An Environment That Permits Calculation: The Sporadically Fed Pool

The third essential simplification is an explicit representation of the chaotic early chemical environment. I have previously defined the sporadically fed pool (Yarus 2012), uncontrolled but nevertheless allowing explicit predictions. A useful geochemical environment (termed a pool) sporadically receives dilute nucleotide inputs. Such substrates arrive at uncontrolled times, but with a constant probability per unit time, and therefore, with exponentially distributed arrival intervals (Yarus 2012). Nucleotide amounts are uncontrolled, approximated as Gaussian distributions (sd = ± 0.5 mean) in response to the suggestion of the Central Limit Theorem for summed variables. All materials have appropriate instabilities, that is, all nucleotides, including the active product, decay at plausible rates (see “Methods”). While one can surely revise details of this implementation of early conditions, the sporadically fed pool is credibly closer to its primordial object than the typical biochemist’s reaction.

While intuition suggests that an uncontrolled environment might obstruct evolution, selected sporadically fed pools instead can be surprisingly creative (Yarus 2016; see “Discussion”).

### Selection of Useful Pool Products: Chance Utility

Pool chemical history is shaped by chance utility (Yarus 2016), a consequence of selection for a sporadically fed pool’s product. Selection of product changes likely pool events. For example, such selection can elect a pool receiving a maximally efficient series of reactants. In one characterized case, such a series ideally supports creation, then replication of an oligonucleotide (Yarus 2013). Such selected behavior can clearly be transient; not necessarily passed to pool descendants.

However, pool selection applied to heterogeneous molecular populations can also change descendant pools permanently, as when an inhibitor is left behind (Yarus 2016) by a successor pool. Such chance utility can be effective within the lifetime of single pools. However, an environment in which multiple nucleotide-containing pools are tested will likely be more productive. Below, effects of selection on such populations are calculated. Chance utility is indispensable because it allows environment-directed modification of purely chemical pools, allowing them to evolve toward inheritance.

### Previous Work on Pooled Self-complementary Dinucleotides

The behavior of self-complementary 5′–5′ coenzyme-like dinucleotides (Yarus 2012) has previously been examined. Replication of such self-complementary dinucleotides seemed a plausible initial hypothesis (von Kiedrowski 1986), given small molecular size and simplified reproduction intrinsic to self-complementarity. Such an RNA model, in fact, appears potentially capable of replication, supposing only already established ribonucleotide rates and capabilities. Further, it could easily change to replicate, even if it did not begin with this property. In that earlier calculation, all molecules were unstable, in order to demonstrate that short lives are not an insuperable bar to biological behavior. Instead, molecules here differ greatly in lifetime, instead of being alike in their instability (“Methods”). Notably, such stability differences themselves comprise potentially productive behavior (below).

### An Explicit Transition to Inherited Chemistry

Under above conditions, inheritance of a novel chemical capability evolves, via known ribonucleotide chemistry, under means plausible in primitive settings. To justify this claim quantitatively, we first define basic pool behavior. Then it is shown that selection strongly stimulates pool template function, using numerical solution (Supplementary Information) of the pool system of differential equations (“Methods”; explicit differential equations are available in Supplementary Information). Selection is then shown to establish templated expression as majority pool behavior.

## Results

### A Concentration Range for Pool Nucleotides

A numerical anchor focusses analysis of nucleotide concentrations. Integrated chemical NppM synthesis (chem) is second order overall **(**Puthenvedu et al. 2015; Majerfeld et al. 2016) and therefore has a rate constant *k*_chem_ with units M^−1^ time^−1^:

1 $${\text{d}}\,{\text{chem}}/{\text{d}}t = k_{\text{chem}} (nt)({\text{activated}}\,nt).$$

Cross-templated NppM synthesis (temp) acts as third order overall at low experimental concentrations for both rA–rU and rG–rC complements **(**Puthenvedu et al. 2015; Majerfeld et al. 2016) and has a rate constant *k*_temp_ with units M^−2^ time^−1^

2 $${\text{d}}\,{\text{chem}}/{\text{d}}t = k_{\text{chem}} (nt)({\text{activated}}\,nt)({\text{template}}).$$

For the G–C system, the above implies that dimer synthesis in templated stacks is equal in rate to that in nucleotide stacks free in solution at a particular template concentration

3 $$({\text{template}}) = \frac{{k_{\text{chem}} }}{{k_{\text{temp}} }} = \frac{2}{1640} = 1.22 \times 10^{ - 3} \,{\text{M}} .$$

Equality in chemical and templated routes occurs when (template) is 1.22 mM nucleotide phosphate. This concentration region is the focus below.

### A Representative Example Pool

**Fig. 1:**
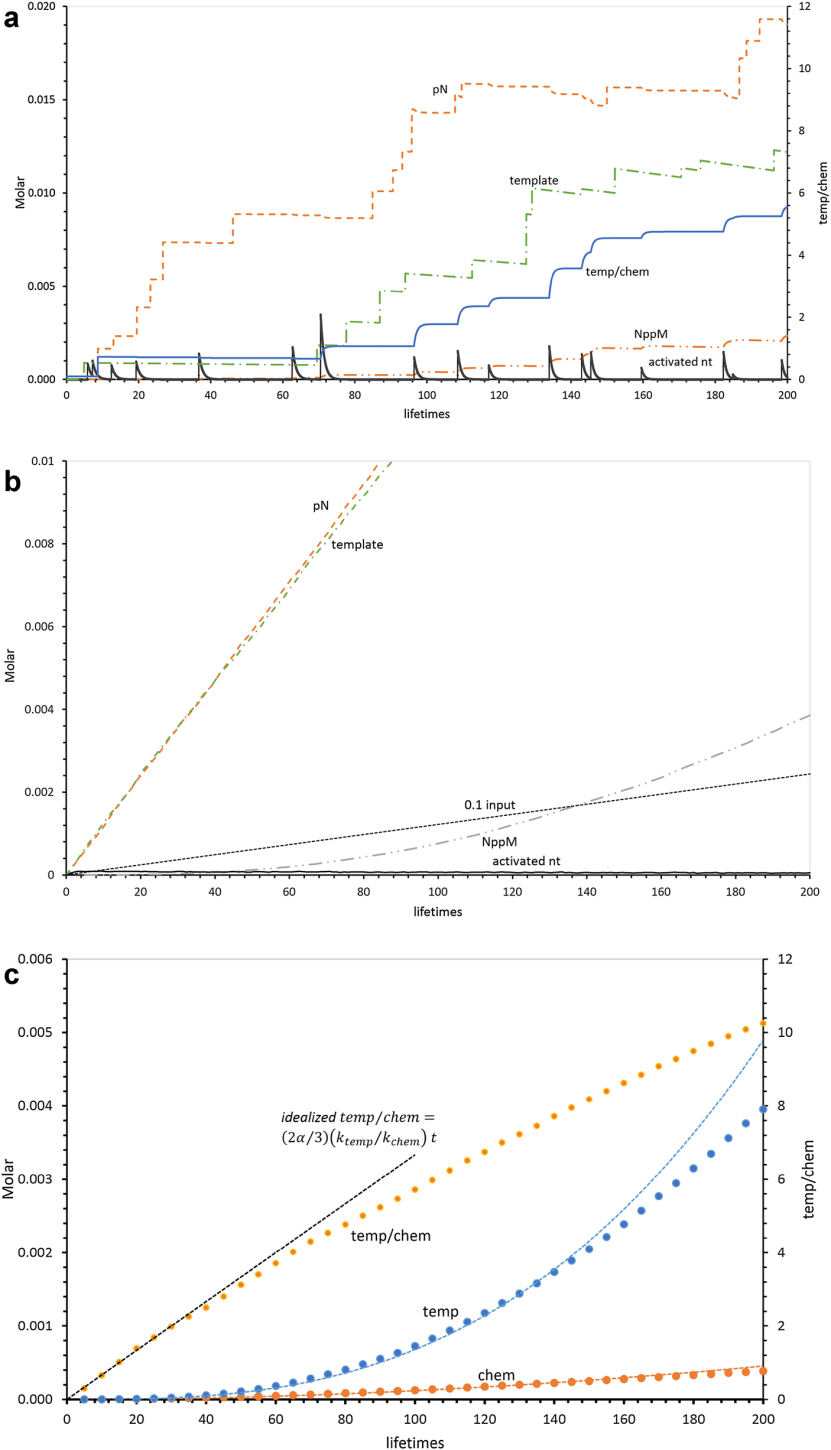
**a** Performance of a representative cross-templating pool. 200 mean imidazolide lifetimes (100 mean lifetimes = about 83 days at 12 °C) in one representative sporadically fed pool of cross-templating ribonucleotides. Reactants reach the pool at random times, at a mean frequency of 10 spikes/100 lifetimes and at a mean spike height of 1.22 mM ± 0.61 mM (SD). The *dashed line* is 5′ pN concentration. The *solid spikes* are the activated nucleotide; with every 100th calculated point shown. The *dash and dotted line* is randomly appearing template polymer. The *dash and double-dotted line* is total 5′–5′ NppM dimer. The *thick solid line* is relative templating, the integrated template-mediated NppM synthesis (temp) divided by total chemical synthesis (chem). These calculations are described in “Methods” and Supplementary Information. **b** Average pool behavior. Average concentrations of reactants in 1000 pools. *Lines* are the same as in Fig. 1a, save for input nucleotide (*dotted line*), which is 10% of mean input for all nucleotide precursors. **c** Chemical and templated synthesis of product NppM. Means of 1000 pools are shown for chemical (chem; Scheme 1) and templated (temp; Scheme 1) routes to NppM product. The fitted *least squares dotted line* accompanying chem points = 1.64 × 10^−8^ lifetime^1.93^. The fitted *least squares dotted line* accompanying temp = 1.34 × 10^−9^ lifetime^2.85^. The* dotted line* tangent to temp/chem is a plot of Eq. 9

One representative pool’s history for 200 mean imidazolide lifetimes is integrated (“Methods”; Supplementary Information) and plotted (Fig. 1a). Activated nucleotide is unstable, so appears as variable randomly occurring spikes which rapidly decay. Sporadic inputs of more stable RNA template and pN accumulate (“Methods”; Table 1), appearing in upper curves as varying near-vertical jumps (pool substrates arrive quickly, in 0.01 lifetime). Importantly, such stable accumulations make the random unstable activated nucleotide spikes (lowest solid line) more effective in synthesis later in a pool’s history.

### Pool Outcome is Shaped by Reagent Accumulations

Nucleotide concentrations were specifically chosen (in Eq. 3) to equalize chemical and templated velocities. However cumulative templating (temp^1^) and cumulative chemical synthesis (chem^2^) are not equal in Fig. 1a—instead temp/chem is 5.55 after 200 lifetimes. Enhanced templated synthesis is an intrinsic pool property, which relies on accumulation of more stable reactants, pN, and template. These are upper curves in Fig. 1; RNA templates and 5′ pN arrive at the same rates (by hypothesis), but more pN collects by chance in this case, and also in general, because pN also is the more stable molecule (Fig. 1b, “Methods”). Vertical jumps mark random reactant arrivals. In contrast, activated nucleotide appears at the same mean frequency as the two more stable molecules, but subsequently decays during the next few lifetimes.

Co-existence of two stable and one unstable nucleotide reactants yields a new pool property. Because stable reactants accumulate, later pulses of unstable activated nucleotide are more efficiently used. In fact, temp/chem shown in Fig. 1a, an index of relative templating, increases after every spike of unstable activated nucleotide. Thus selecting more product NppM would also select templating—greater amounts of product increasingly are templated molecules.

### Likely Pool Behavior: The Average Pool

Often we need probable pool behavior, rather than a variable single example, as shown in Fig. 1a. The mean behavior of 1000 pools like that in Fig. 1a is shown in Fig. 1b.

Stable reactants, pN, and template, increase roughly linearly when erratic inputs to individual pools are averaged. pN is eventually slightly more abundant because it is somewhat more stable (“Methods”). In contrast, an unstable activated nucleotide, on average, is present at a low, non-increasing mean concentration because, usually, it decays before the next such input arrives (Fig. 1a). Product NppM (dashed, Fig. 1b) increases very non-linearly, because its synthetic rate increases as the product of two linear increases (pN and template) and a rough constant (activated nt).

### Mean Early Pool Accumulations can be Calculated

Mean pool accumulations (Fig. 1a, b) can be understood more generally. Envisage an idealized early pool in which nucleotide decays are negligible because time has been too short for decay. Consumption of nucleotide via small amounts of synthesis is also still negligible with respect to nucleotide supplies. This approach gives rise to a serviceable approximation for pN and template polymer in early pools. If nucleotide supplies (*nt*) arrive at an average rate (M/time), then (despite underlying individual randomness in supply) averaged stable nucleotide increases as

$${\text{d}}(nt){\text{d}}t = \alpha - k_{\text{d}} (nt).$$

This differential equation includes the mean rate of nucleotide appearance (***α***) and the rate of subsequent decay (*k*_d_). This can be solved for time *t* to get

$$(nt) = (\alpha /k_{\text{d}} )(1 - {\text{e}}^{{ - k_{\text{d}}t}} ).$$

So for stable reactants (*k*_d_ is small), ${\text{e}}^{{ - k_{\text{d}}t}} \approx{1}-{{k_{\text{d}}t}}$, and:

$$(nt) \cong \alpha t.$$

Thus, plausibly, stable reactants increase linearly at close to the rate they are supplied (Fig. 1b). Almost stable reactants will increase a bit less than linearly.

In contrast, unstable reactants (first-order decay *k*_d_ is large, decay rapid), like activated nucleotide, behave differently. Their concentration(s), (nt), rise exponentially over several *k*_d_^−1^ to

$$(nt) \cong \alpha /k_{\text{d}}.$$

Thus average activated nucleotide, instead of accumulating indefinitely, increases to approximately *α*/*k*_d_, until synthesis becomes large enough to consume it. Here, *α*/*k*_d_ = 1.22 × 10^−4^ M, visible as “activated nt” running across the bottom of Fig. 1b.

Thus, early in pool life, idealized averaged reactants are all known:

4 $$nt \cong at,\quad {\text{template}} \cong at,\quad {\text{activated}}\,nt \cong \alpha /k_{\text{d}}.$$

Because the chemical and templated reactions have known rates (Majerfeld et al. (2016); Eqs. (1) and (2) above):

5 $${\text{d}}\,{\text{chem}}/{\text{d}}t = (nt)({\text{activated}}\,nt) \cong \sim k_{\text{chem}} \alpha^{2} /k_{\text{d}} t$$

6 $${\text{d}}\,{\text{temp}}/{\text{d}}t = (nt)({\text{activated}}\,nt)({\text{template}}) \cong \sim k_{\text{temp}} \alpha^{3} /k_{\text{d}} t^{2}.$$

Integrating these to get chem and temp as a function of early times:

7 $${\text{chem}} \cong (\alpha^{2} /2k_{\text{d}} )t^{2}$$

8 $${\text{temp}} \cong (\alpha^{3} /3k_{\text{d}} )t^{3}$$

9 $${\text{temp/chem}} \cong (2\alpha /3)(k_{\text{temp}} /k_{\text{chem}} )t.$$

**Fig. 2:**
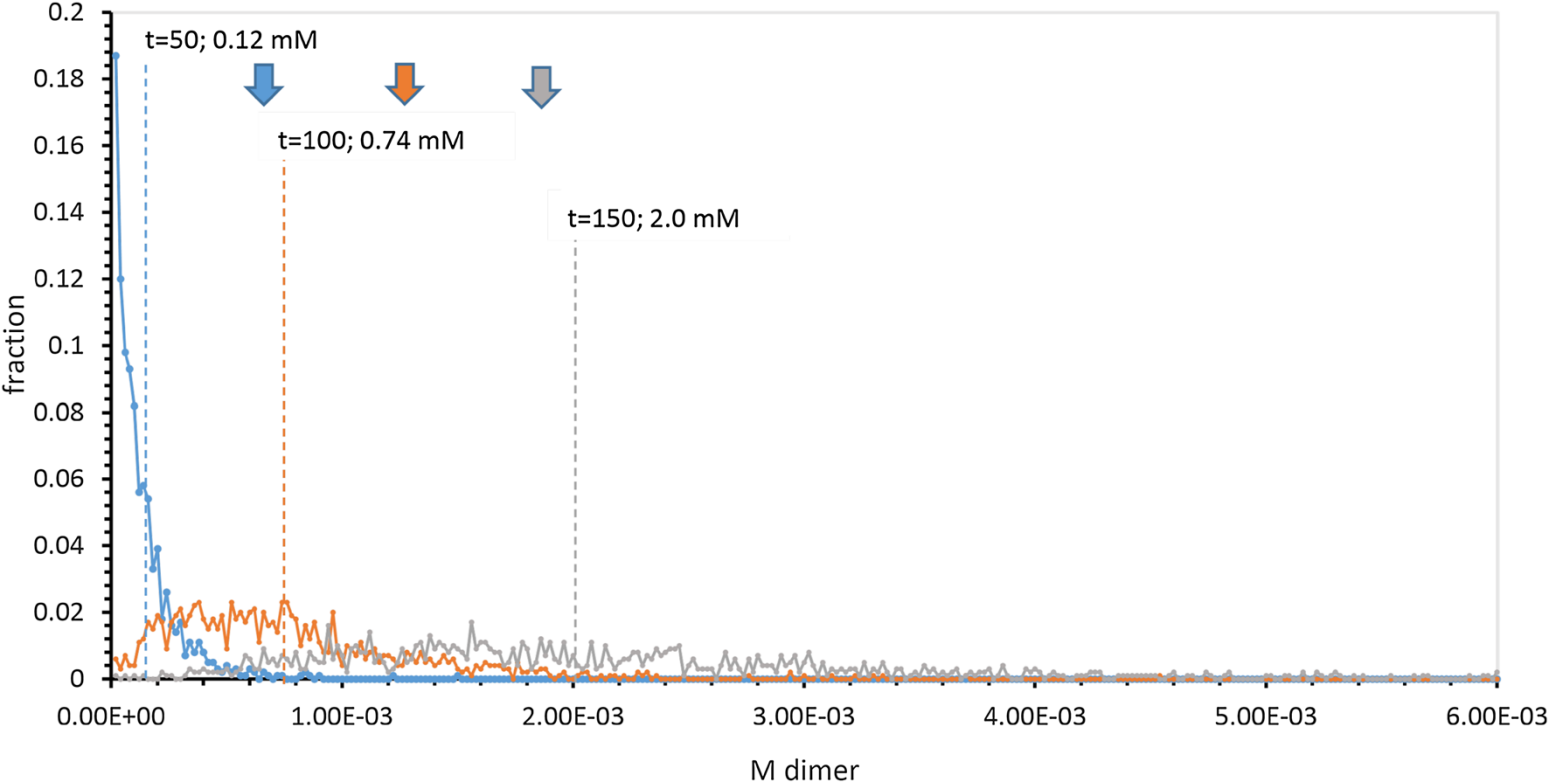
Histogram for NppM output from 1000 pools subjected to periodic hazard, that is, incubated to uniform times of 50, 100, and *t* = 150. *Colored arrows* at *top left *indicate 0.1 × mean nucleotide inputs for, *left* to *right* 50, 100, and *t* = 150 (means of 0.5, 1, and 1.5 nucleotide spikes). *Dashed vertical lines* mark the calculated means (expected value) for observed NppM distributions

So, in early pools, we predict time-squared dependence of mean chem (Fig. 2b; Eq. 7), time-cubed dependence of average templated NppM (Fig. 2b; Eq. 8), and thus also a linear increase of relative mean pool templating with time (Eq. 9). Time-cubed increase in templated output and thus increasing temp/chem are crucial properties during pool selection below.

### Templating Increases with Time

Figure 1c shows that the above averaged expectations (Eqs. 7–9) are obeyed early on, even in a more complex pool environment that includes decays and NppM synthesis. Figure 1c shows cumulative chemical (chem) and templated (temp) synthesis, alongside the cumulative ratio (temp/chem), our index for relative templated NppM synthesis. Least squares dotted lines fitted to calculated data show that templated NppM production is approximately proportional to time-cubed (∝ time^2.85^; see the Fig. 1c legend). Chemical synthesis of NppM, instead, increases approximately as time-squared (∝ time^1.93^; see legend). Thus early relative templating is increasing linearly, proportionate to lifetime (idealized temp/chem in Fig. 1c; Eq. 9) until the consumption of precursors in synthesis and decay reduces real NppM output below the idealized level.

### Pools Can Make Products in the Same Order as Nucleotide Supplies

We call pools “efficient” if they make products of the same order of concentration as their precursor nucleotides. Figure 1b shows that average pools can be efficient. The dotted line in Fig. 1b is 0.1 averaged nucleotide input of 1.22 mM/10 lifetimes. Mean NppM output crosses this line, into the order of nucleotide input at 138 lifetimes. Thereafter, the average pool is efficient. We will refer to this idea of efficiency repeatedly below.

Such efficiency is related to a previous observation. It has been noted that pools accelerate synthesis during accidental superposition of randomly arriving, unstable substrate spikes (Yarus 2013). Here we extend this notion to more stable reactants, which necessarily superpose because they persist as summed reactant concentrations.

Finally, this discussion of Fig. 1a, b illustrates the present method of argument—the chaotic behavior of individual pools clearly displays underlying events (e.g., Fig. 1a), but anticipated pool behavior (e.g., Fig. 1c) is best reflected in averages from multiple pools. Thus, reliable average and chaotic individual pool properties are both considered.

### A Provisional Lower Nucleotide Limit for Pool Templating

Templating does not become the major mode of synthesis for a similar pool, but supplied with nucleotides an order more dilute, that is, similar but with nucleotide inputs of 0.122 mM. This observation is somewhat arbitrary; for example, relative templating varies with different substrate arrival schedules, or under selection (below). Nevertheless, in view of these data, I will cite 10^−3^ M nucleotides as a provisional lower limit for efficient templating.

### How Pool Synthesis Evolves Under Selection for Product

Data above already suggest a compelling inference about evolution of cross-templating pools. Selection for a useful product, at any point in average pool history (Fig. 1b), apparently impels an average pool from chemical synthesis, toward reliance on a gene-like complementary template (rightward in Fig. 1a, c). While this remark relies on average pool properties, and on a simplified selection, this interesting progress to templating reemerges when such simplifications are remedied below.

### Pools Live Limited Lives

Pool life history is crucial to selection. Here we distinguish two kinds of history, with widely differing effects.

### Pools Subjected to Periodic Hazards

On the one hand, cyclic events can limit pool lifetimes. Here we envision recurring hostile environments, making pool survival some multiple of an underlying cycle time. For example, pool lifetime may be coupled to day–night temperature variation or to tidal events that regularly inundate a pool. We will call these cases examples of *periodic hazard*. Pools perishing from periodic hazard have lifetimes which are a multiple of their cycle times. For example, pools below perish at *t* = 50, 100, or 150.

### Pools Subjected to Constant Hazards

On the other hand, there may alternatively be a constant chance of pool dissolution per unit lifetime. This would correspond, for example, to disruption by an impactor that melts the environment of a pool. Or, perhaps such disruption occurs via rain- or snowfall, which with roughly constant probability through time, dilutes pools and stops reactions. We will call such pool fates examples of *constant hazard*. Constant hazard pools have varied lifetimes that, for comparisons, will average to equal periodic lifetimes. For example, pools might perish at *t* = 〈100〉, when exponentially varied pool lives average to 100.

### Pool Output Under Periodic Hazard

In Fig. 2, expected pool outputs (dashed vertical lines) demonstrate rapidly increasing average dimer output with time, as previously seen in Fig. 1b. One-tenth average nucleotide inputs are marked by filled downward arrows at the top left. Pools to the right of respective filled downward arrows, therefore, make products of the same order as nucleotide inputs. Comparison with mean output (vertical dashed lines) shows that average periodic hazard pools approach and then enter the order of their inputs. In detail, 1.1% (*t* = 50; mean 1.2 × 10^−4^ M), 16% (*t* = 100; mean 7.4 × 10^−4^ M), and 47% (*t* = 150; mean 2.0 × 10^−3^ M) of periodically threatened pools are efficient. Thus pools receiving 1.22 mM nucleotide inputs make efficient use of dilute supplies, consistent with previously cited mean pool behavior, which becomes efficient at *t* = 138 (Fig. 1b).

### Pool Output Under Constant Hazard

**Fig. 3:**
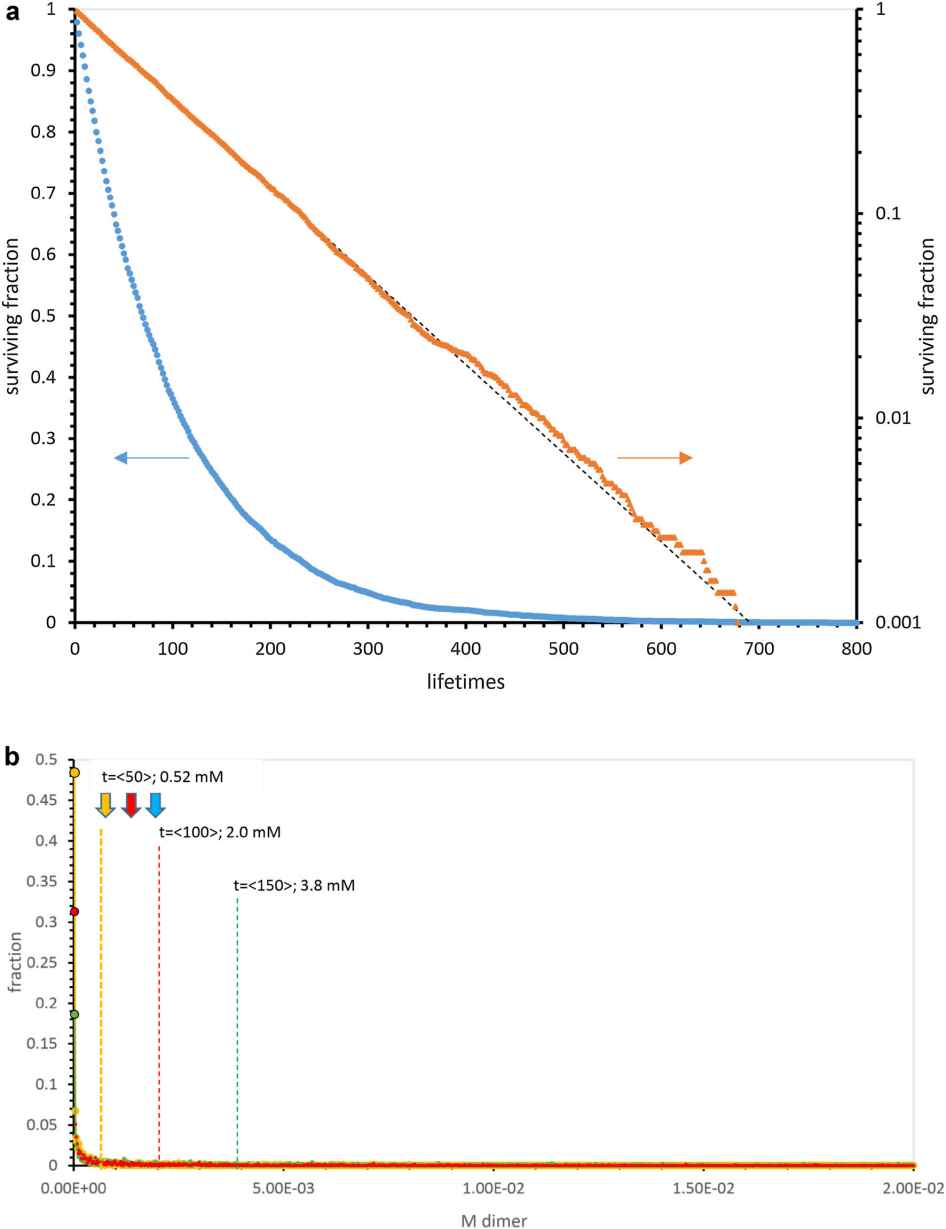
**a** Distribution of lifetimes in a constant hazard pool with a mean lifetime *t* = 〈100〉. Frequencies among 1000 pools surviving at each lifetime are plotted linearly (*circles*; *left axis*) and logarithmically (*triangles*; *right axis*). The dashed line marks perfect exponential behavior, e^− time/100^. The computed mean ± SD of these generated exponential lifetimes (“Methods”) are 100.0 ± 100.4, in excellent agreement with theory for exponential lives: 100.0 ± 100.0. **b** Histogram for coenzyme-like output from 10,000 pools subjected to constant hazard, with mean lifetimes = 〈50〉, 〈100〉 and 〈150〉. *Filled arrows * at the* top left* indicate 0.1 × average nucleotide input from 0.5, 1, and 1.5 spikes of 1.22 × 10^−3^ M nucleotides *left* to *right*. *Vertical dashed lines* show the calculated mean (expected) total dimer from each distribution. The bin containing lowest NppM output (*large circles *on* leftward ordinate*) contained 48% (〈50〉), 31% (〈100〉), and 19% (〈150〉) of pools

Pools that die with constant probability exhibit a strikingly different product distribution (Fig. 3b), compared to periodic hazard. Because constant hazard produces an exponential lifetime distribution (Fig. 3a), many constant hazard pools die early. Constant hazard populations therefore contain many entirely unproductive pools (Fig. 3b legend). Failed pools are plotted as enlarged points at the leftward axis, decreasing as pools age. But conversely, exponentially distributed lives also imply a significant tail of very long-lived, productive pools (Fig. 3a, b), extending with longer life, and with significant numbers out to several times the mean lifetime of 〈50〉, 〈100〉, or 〈150〉.

Thus, Fig. 3b shows advancing mean production with mean life: with 1.22 × 10^−3^ M nucleotide spikes, we see 5.2 × 10^−4^ M 〈50〉, 2.0 × 10^−3^ M 〈100〉, and 3.8 × 10^−3^ M 〈150〉 expected output. Downward filled arrows (upper left) again mark 0.1 × nucleotide input, left to right. Thus constant hazard pools are frequently efficient at all these times—20% efficient at 〈50〉, 29% efficient at 〈100〉, or 35% efficient at 〈150〉. As also with periodic hazard just above, constant hazard cross-templating pools receiving 1.22 × 10^−3^ M spikes frequently make products of the same order of magnitude as input nucleotides. In fact, because of rapid increase in output with pool age (Eq. 8; Fig. 1b), both mean output and efficiency of constant hazard pools are increased (versus periodic hazard), because of the constant hazard’s characteristic flat, long tail of very productive pools. Pools in these tails (Fig. 3) benefit greatly from mean time-cubed templating (Fig. 1c; Eq. 8). Thus constant hazard pools have greater mean outputs (compare periodic pools above) in spite of frequent barren examples (large ordinate points, Fig. 3b).

### Mild and Strong Selection for an NppM Pool Product

**Fig. 4:**
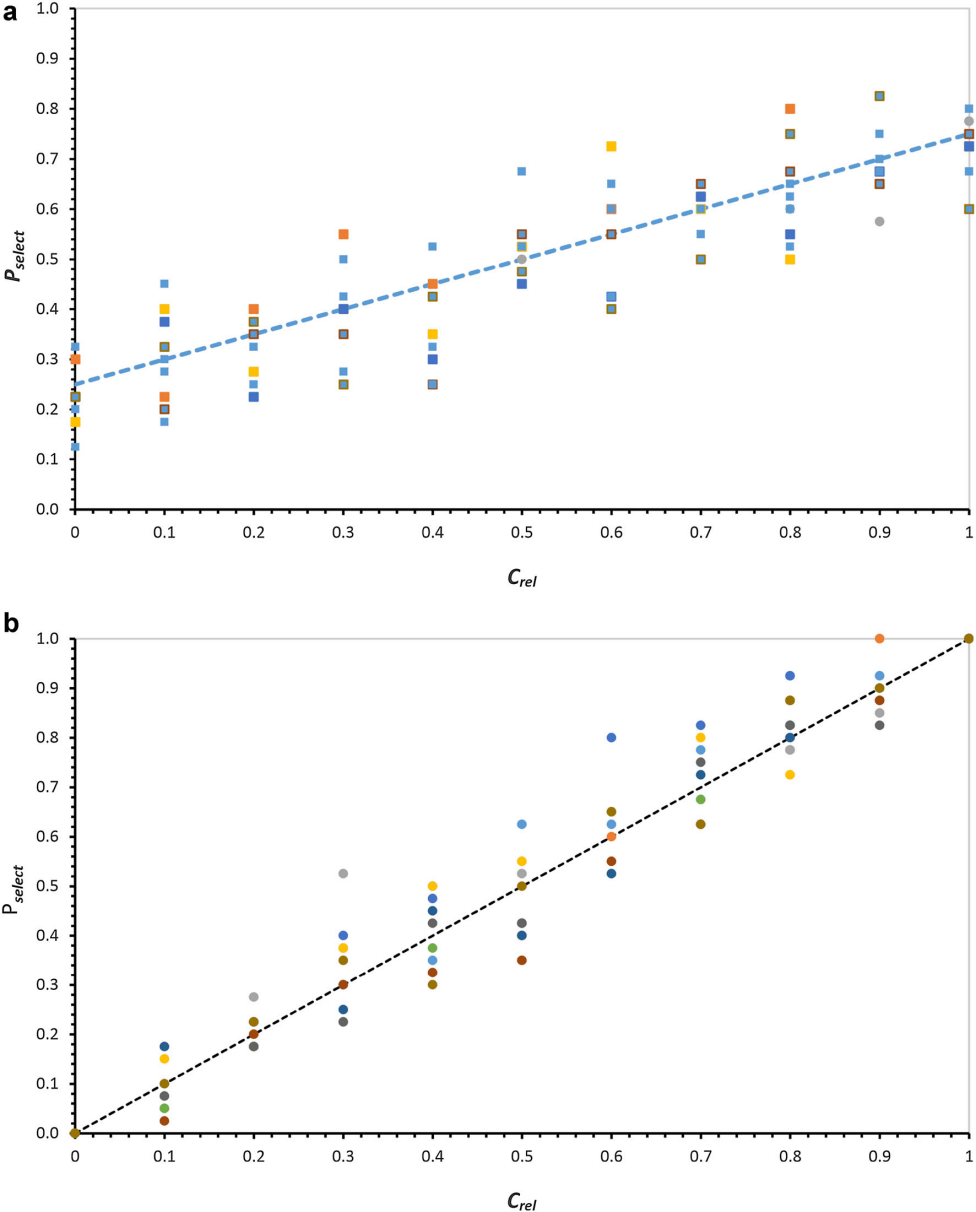
**a** Mild selection for product. The dashed line plots probability of selection with product concentration, *P*_select_ = 0.25 + 0.5*C*_rel_ where *C*_rel_ = *C*/*C*_max_, its ratio to the maximum observed value. The circles are 440 simulations of selection at this probability as Bernoulli trials, to illustrate variation in actual selections conducted at the same underlying probability. **b** Strong selection for product. The dashed line plots probability of selection with product concentration, *P*_select_ = *C*_rel_. The circles are 360 simulations of selection at this probability as Bernoulli trials, to illustrate variation in actual selections given the same underlying probability

There are many ways to implement selection, and here we do not intend an exhaustive study. The crucial idea is that NppM is useful, that is, probability of selection increases as pool gene product concentration increases. Most simply, selection probability increases linearly with NppM concentration. This notion is used in two forms: mild selection (Fig. 4a), in which the transition from no product to its maximal concentration linearly increases the probability of pool selection, *P*_select_, from 0.25 to 0.75. And strong selection, in which the probability of selection with no gene product is zero, and the probability of selection with maximal levels of product is one (with linear increases in between (Fig. 4b). The selection probability is plotted (dashed lines) versus the relative product concentration, *C*_rel_, as shown in Fig. 4a(mild), b(strong).

Selections of individual pools are so-called Bernoulli trials; they can succeed or fail. With a probability of selection of *P*_select_, actual selection exhibits a standard deviation of $\sqrt {n(P_{\text{select}} )(1 - P_{\text{select}} )},$ where *n* is the number of selections. To illustrate this expected variation, selection was carried out in 10 sets of 40 at each *C*_rel_, and these 10 observed probabilities are plotted in Fig. 4 as circles for each relative product concentration. Under mild selection, pools with no product are frequently nevertheless chosen, and pools with maximum product are nevertheless frequently eliminated. In contrast, strong selection determines that maximum concentrations will survive, and minimal ones rarely do.

Selections are called mild and strong in view of the fraction of pools which survive. Mild selections accept a quarter to a third of pools. Strong selections restrict pool population survivals more impressively, one in 50 to one in six proceed. Mild and strong selections as defined (Fig. 4) have the same mean probability of selection (0.5), but differ in slope. Accordingly, comparison of the two can be roughly summarized as an inquiry into the effect of d*P*_select_/d*C*_rel_, the rate of change of selection probability with change in relative product concentration.

### Selection Acts on Populations of Individual Pools

To apply mild (Fig. 4a) and strong selection (Fig. 4b), we take periodic hazard (Fig. 2) and constant hazard (Fig. 3) pool product distributions and apply the probability of selection suggested by the *C*_rel_ of each pool. This yields a new, selected distribution—roughly speaking, normalized pool frequencies will be depressed by low *P*_select_ at low *C*_rel_, nearly unchanged in the middle, and then elevated at larger *C*_rel_ (Fig. 4). In other words, a selected distribution is shifted to higher mean product concentrations.

### Selection at Different Pool Ages

**Fig. 5:**
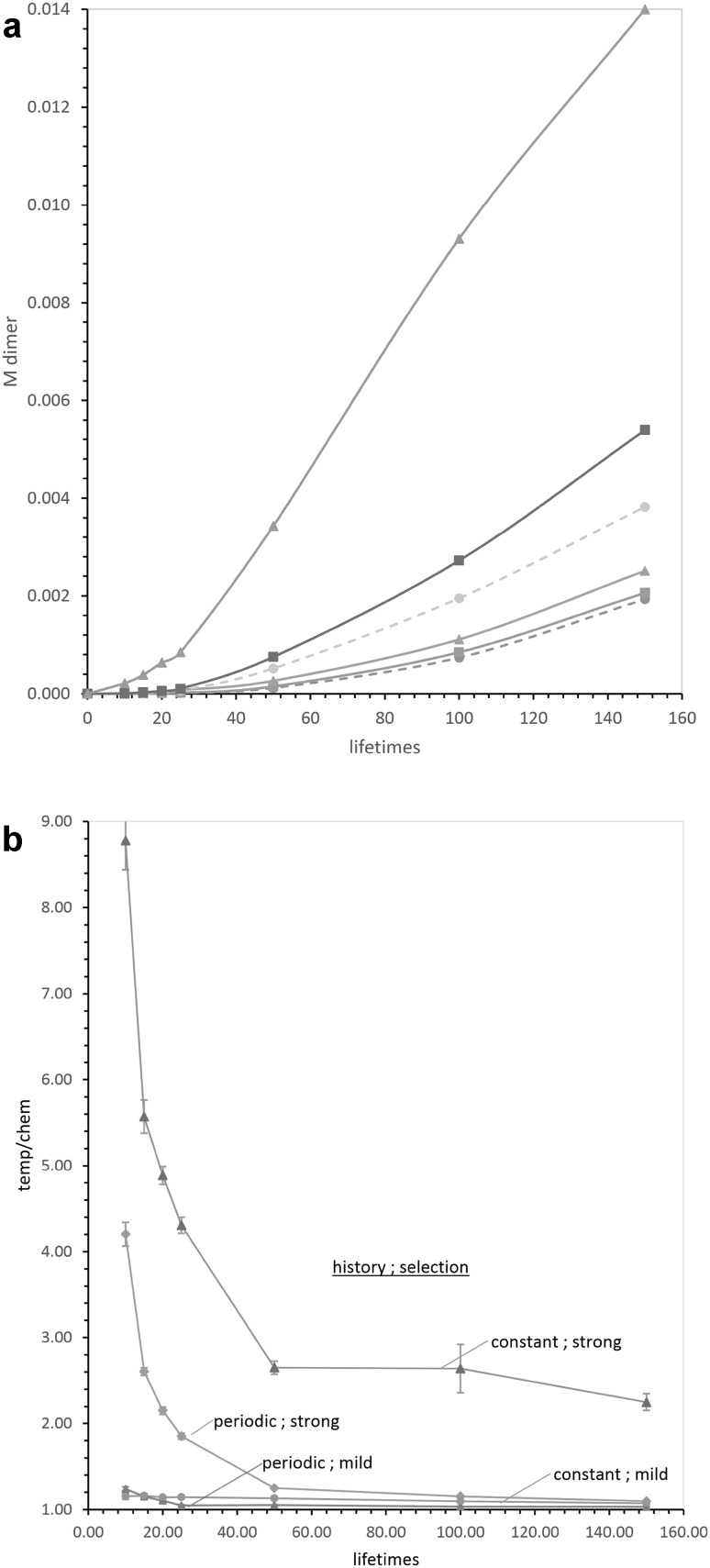
**a** NppM output increases with time and selection. *Dashed lines* and *circles* refer to unselected pools; *solid lines* to selected ones. *Squares* and *solid lines* are mildly selected, and *triangles* are strongly selected. *The lower triplet of lines* are periodic hazard pools, *the upper triplet* constant hazard. All *points* are means of 25 trials with 1000 independent pools. **b** Relative templating (temp/chem) increases disproportionately under selection in both pool types, at all pool ages. Δtemp/chem is (temp/chem after selection) normalized to (temp/chem before selection). *Triangles* are constant hazard pools under strong selection; *circles* are constant hazard pools under mild selection; *diamonds* are periodic hazard pools under strong selection; *triangles* are periodic hazard pools under mild selection. All *points* are the means of 25 repetitions using 1000 example pools; *error bars* are sem. Where sem *bars* are invisible, they are within points

Pool selections are summarized in Fig. 5a, which quantitates mean pool output in both periodic and constant hazard pools, and under varying selection and pool ages. The salient points are that selection on output is seen at every pool age and age distribution—but the same selection, whether mild or strong, is much more effective in constant hazard pools. Thus, after selection on a constant hazard pool at 〈50〉, output is elevated 1.5-fold by mild, and more than sixfold by strong selection. Accordingly, selected pools at any age become, on average, markedly more efficient.

### Effect of Selection on Relative Templated Expression, Temp/Chem

Results in Fig. 5a show that mild and strong product selection increases pool production and efficiency, consistent with intuition. However, this is less relevant to evolution than is selection for increased templating, as expressed by temp/chem. Temp/chem is plotted in Fig. 5b, which plots the change in templating (Δ temp/chem) under selection—determining the index for the selected pool, then normalizing to temp/chem for the prior, unselected pool. This calculation is shown for pools under selection at times from 10 to 150, reasoning that these times cover a complete transition from early small, idealized pools (Fig. 1b) to later synthesis-with-significant-decay and nucleotide consumption (Fig. 1c) and slower later increase in temp/chem (Fig. 1b).

The effects of selection on templating (Fig. 5b) are particularly striking. All kinds of pools, and at all ages, increase relative template activity (temp/chem) in response to selection, but constant hazard pools are usually more responsive than periodic hazard pools. Moreover, strong selection is markedly more effective early, accelerating early pools toward templated synthesis.

Differences attributable to pool history can be large. For strong selection on constant hazard 〈50〉 pools, temp/chem changes 2.7-fold in a single cycle of strong selection, and 1.13-fold under mild selection. The corresponding numbers are 1.25- and 1.05-fold for periodic hazard. Thus the constant hazard pool responds more strongly. This can be viewed as a quantitation of chance utility (Yarus 2016), exemplifying a change in the preferred route to pool product under selection. However, little selection would be required in any of these pools to complete a transition to template usage (Table 1; quantitated in “Discussion”).

Selection also responds disproportionately to in d*P*_select_/d*C*_rel_, representing severity of selection. At 50 lifetimes in periodic pools, temp/chem increases about fivefold more for strong than mild selection. But for constant hazard pools at *t* = 〈50〉, the increase in Δ temp/chem from mild to strong selection is about 20-fold. This finding is itself of great potential interest; this is a co-operative nucleotide stacking system (Majerfeld et al. 2016) and co-operative phenomena show very rapid changes in properties near their transition midpoints. Therefore, extremely rapid selection of templating due to large d*P*_select_/d*C*_rel_ as a result of co-operative change in stacked ribonucleotide properties is worth further thought.

### Constant Hazard Pools are Superior Under Selection

Constant hazard pools are more responsive to selection (above) because constant hazard pools include older and more productive members. One way to see this is to calculate mean ages of selected pools. Selected 〈50〉 pools have mean ages of 57 lifetimes (after mild selection) and 165 lifetimes (after strong selection). Selection, roughly speaking, constructs an older population of pools with accompanying age-dependent increases in mean output and templating (Fig. 1b, c).

**Fig. 6:**
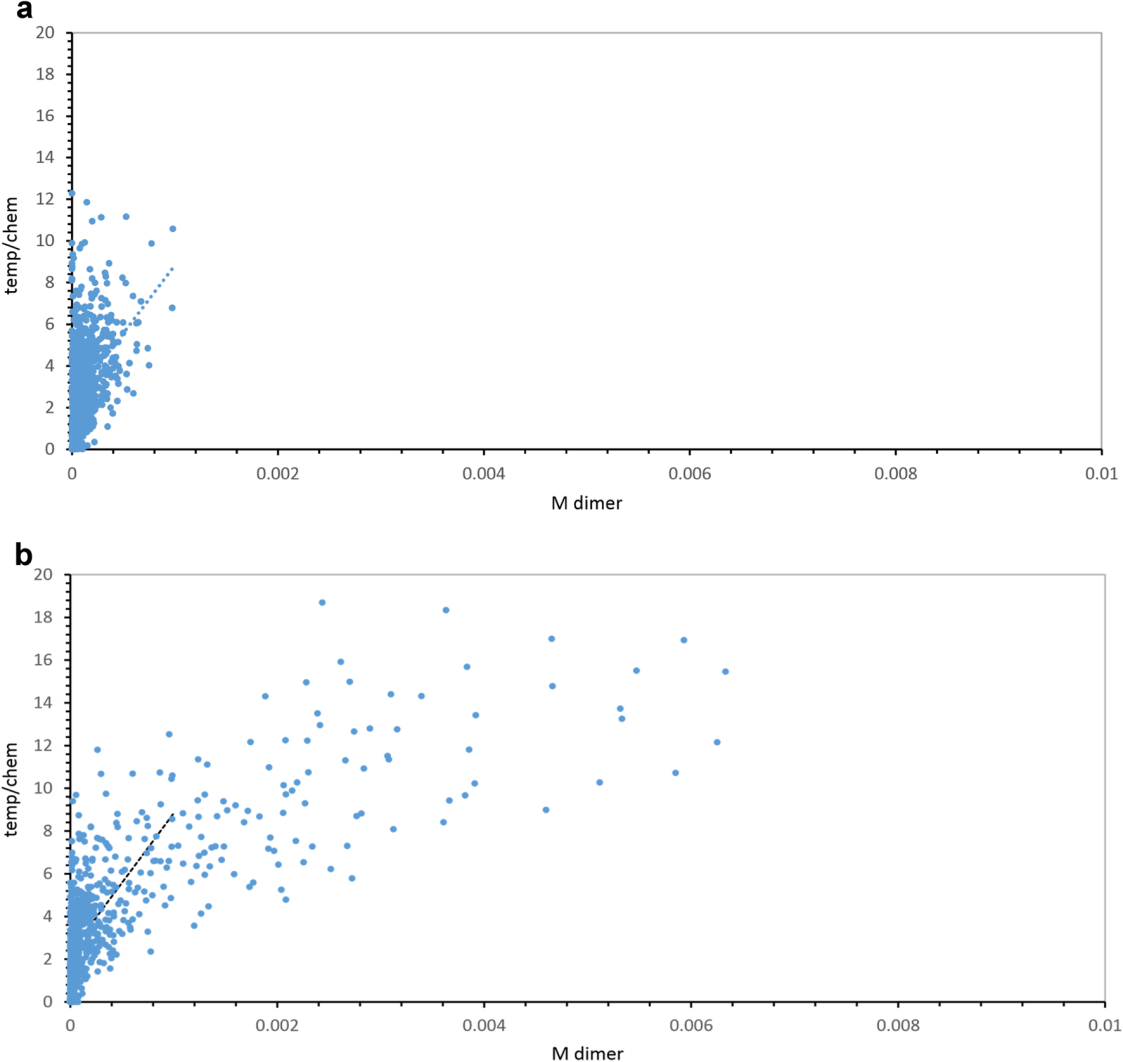
**a** Relative templating versus output, periodic hazard pools, *t* = 50. Cumulative templated/chemical output is plotted versus total NppM produced by the same pool, for 1000 unselected pools. The *dashed line* is a linear least squares fit to early temp/chem versus NppM. **b** Relative templating versus output, constant hazard pools, *t* = 〈50〉. Cumulative templated/chemical output is plotted versus total NppM produced by the same pool, for 1000 unselected pools. The *dashed line* is from (**a**)

More precisely, selection in periodic hazard pools acts only on differences due to stochastic substrate and template amounts and arrival. Constant hazard pools also have these factors in full measure, plus the strong variation in output that is due to varied pool lifetimes (Fig. 2; Eqs. 7, 8). These differences between pools with different histories are visualized in Fig. 6a, which shows temp/chem in 1000 unselected periodic hazard pools, all age 50 (points), plotted against NppM output of the same pool. The accompanying line is the least squares fit to 1000 points.

Thus, even in these periodic hazard pools, where product is made at low concentrations, templating is favored when more NppM output is demanded (points and line, Fig. 6a). Though these pools have identical ages, more productive pools (greater NppM) have had greater template function (greater temp/chem).

Figure 6b is similar, but for the matched constant hazard pool at mean age 〈50〉. The least squares line from Fig. 6a is reproduced for comparison.

The distribution of pools near the dashed line is similar. Thus periodic and constant hazard pools differ only slightly for low outputs. But a constant hazard pool (Fig. 6b) has a greatly extended upper limb with high NppM output, corresponding to the average initial time-cubed increase in templated output (cf. Eq. 8), and the accompanying increased templating, seen as temp/chem (Fig. 1c; cf. Eqs. 7–9). Thus selection for output and temp/chem in constant hazard pools is more effective because it accesses the characteristic extended upper tail (see Fig. 3) of high, mostly templated, constant hazard pool outputs. This upper tail, in turn, is the result of reactant accumulation and consequent increasing pool output which begins as a power of pool lifetime (Fig. 1c; Eqs. 7–9).

Any change in pool history that shifts a periodically hazardous environment toward one allowing varied pool lifetimes therefore speeds evolution of templating behavior. Pools within mixed environments might be winnowed, those with varied lifetimes becoming dominant because of their more rapid evolution, even if mean lifetime is similar for all pools.

### Selection Does Not Require Thousands of Pools

Evolution does not require that primordial nucleotides be divided among thousands of separate pools. Large samples are required to accurately define rapid changes in time (as in Fig. 3), or to accurately define the smaller numerical effect of selection with periodic hazards (as in Fig. 5). However, these observational requirements do not imply that the underlying mechanisms would vanish for a few pools, as may be more likely in a primordial environment. Indeed, selection has the same mechanism and ultimately the same effect in five pools as in 5000. Moreover, pool populations under selection can be successive, separated in time rather than multiplied across a geochemical landscape.

### These Calculated Selection Effects are Minimal

Computed changes in templating resulting from a single cycle of selection (Fig. 5b), are highly significant—but they are, in fact, minimal estimates. These pools are populated by uniform molecules performing identical reactions. Such a homogeneous system allows optimization of selected chemical events, but not a permanent structure-altering choice between chemistries, for example, as for the poison exclusion example of chance utility (Yarus 2016). For a second parallel reaction, either faster or slower than the modeled one, selected pool changes could be larger and more permanent. For example, a less active system might be eliminated. Thus, quantitative differences reported (Figs. 5, 6) are in an important sense, lower limits.

## Discussion

Rates here are based on laboratory results for 5′–5′ purine dimer synthesis from 5′––3′ poly(C) template (Majerfeld et al. 2016; see “Methods”). Kinetic calculations (“Methods”; Supplementary Information) were combined with these data to assess likely primordial outcomes, on the basis of standard chemical kinetics. For example, we computed effects of first-order molecular decays (Yarus 2012; “Methods”) and of erratic availability of nucleotide substrates. The major result is that pooled cross-templated RNA reactions under selection are not only logically sufficient (Majerfeld et al. 2016), but also kinetically sufficient (e.g., Figs. 5b, 6b; Table 1) to establish a primitive genetic circuit.

### Efficiency of Chaotic Supplies to a Sporadically Fed Pool

This kinetic inquiry (Figs. 1, 2a, et seq) points to unanticipated competence in a pool of cross-templating ribonucleotides. For a real cross-templating ribonucleotide system (see “Methods”), such a pool is not only efficient in production of its product, but also quite robust to environmental variation. Intermittent millimolar concentrations of reactants, including unstable as well as relatively stable molecules, are more than sufficient, in a process with a lifespan of months. Such a pool frequently produces products of the same order as its precursors. This efficiency grows markedly more common as pools age (Figs. 2a, 3b), and is average pool behavior at *t* = 138 lifetimes (Fig. 2b). Moreover, efficiency is highly responsive to selection. Even early, minimally productive *t* = 〈10〉 pools become 32% efficient under strong selection (Table 1). The sporadically fed pool is an unexpectedly capable chemical reactor, rapidly more efficient with pool age (Eqs. 7, 8; Fig. 1b, c) and continuously increasing emphasis on a templated gene product (Eq. 9; Figs. 1c, 5), especially under selection (Table 1).

### Quick Evolution Toward Templating

Evolution to simple inherited chemistry can be quick. Given the combined effects of pool history (Eq. 9; Fig. 1c) and selection (Table 1), pools produce mostly templated NppM after one selection cycle at all pool ages. This quick progress requires intrinsic pool productivity, augmented by product selection. To quantitate this, a modification of our templating index is convenient. Temp/chem is advantageous because of its intuitively transparent comparison of two critical pool activities. But to discuss ultimate outcomes, temp/chem can be related to *f*_temp_, the fraction of templated NppM synthesis:

10 $$f_{\text{temp}} = (1 + {\text{chem}}/{\text{temp}})^{ - 1} .$$

**Table 1:** Summary: selection plus pool dynamics deliver templating in all pools

Selection	Pool history	Early pool example			Late pool example		
		10 lifetimes			150 lifetimes		
		% eff	temp/chem	*f*_temp_	% eff	temp/chem	*f*_temp_
Unselected	Periodic hazard	0	0.277	0.217	47	8.73	0.897
Mild select		0	0.343	0.255	51	8.97	0.900
Strong select		0	1.16	0.538	68	9.60	0.906
Unselected	Constant hazard	1.6	0.380	0.275	35	9.38	0.904
Mild select		2.8	0.437	0.304	41	11.6	0.921
Strong select		32	3.34	0.769	97	19.8	0.952

### Early Pools Allow Effective Selection

In unselected early pools (10 lifetimes, Table 1), templating is the minor synthetic route. At these early times, before decay and significant synthesis, NppM output is also small with respect to available nucleotide levels (Table 1; Fig. 1b). This is true in both periodic and constant hazard pools (Table 1). Notably, mild selection in early pools increases templating, but strong selection makes templating dominant for both pool histories, though neither unperturbed pool favors templating (cf. temp/chem, *f*_temp_, Table 1).

Temp/chem in early pools responds disproportionately to d*P*_select_/d*C*_rel_. The twofold increase in d*P*_select_/d*C*_rel_ from mild to strong selection (Fig. 4a, b; Table 1), greatly enhances both NppM under selection (Fig. 5a) and also enhances relative templating (Fig. 5b). Pool histories are crucially important. Increase in temp/chem in a 10 lifetime periodic hazard pool is 13-fold greater after strong than after mild selection (Table 1); for constant hazard, increase in temp/chem is 50-fold greater after more stringent selection.

In early pools, chemical output (chem) is increasing with near the square of time (Eq. 7; Fig. 1c), alongside templated output (temp) increasing with near the cube of pool lifetime (Eq. 8; Fig. 1c). Thus early pools change rapidly. Under these conditions, with 1.22 mM nucleotides, cross-templated synthesis quickly increases to become the dominant route to NppM (around 20 lifetimes; Fig. 1c; Table 1), even though mean temp/chem is zero at pool origin (Fig. 1c).

### Late Pools Efficiently Produce Product

Late pools (150 lifetime data in Table 1) have passed beyond early power-of-time dependencies (Fig. 1c; Eqs. 7–9), and, aided by their associated substrate accumulations (e.g., Fig. 1a), possess elevated NppM (Figs. 2, 3), high efficiencies, large temp/chem, and associated high templating (Table 1). In fact, all late pools, selected and unselected, periodic and constant hazard, are mostly templating (Table 1). Late templating is therefore uncoupled from selection. In fact, unselected late pool replicators can show slight or no function. The fraction of templated synthesis (*f*_temp_) can change only slightly, perhaps insignificantly, even under strong selection (Table 1).

Therefore, despite quantitative and templating excellence, late pools are unproductive under selection, chosen only if they accidently include functional molecules. Moreover, late pools are by definition slow to present phenotypes, and likely to be outrun by early pools. Thus, we expect selected early pools to first exhibit useful encoded functions.

### An Optimal Pool Succession, Early to Late

Envision a pool selected for function early, thereby benefitting from early pool selection superiority (Fig. 5b; Table 1). Afterward, under selection or not, it survives to have a late pool’s quantitative and efficiency advantages (Fig. 1b, c; Table 1). Survival is especially plausible, because by premise, this early pool deploys a selected advantageous product. Thus, its successor late pool not only makes a selected advantageous product, but in abundance (Table 1). Early-to-late succession therefore creates a particularly potent progenitor for further evolutionary development.

### Selection of Sporadically Fed Pools Gives Unexpected Results

These selected outcomes are surprising. Before analysis, it would seem intuitive that sporadic, unstable substrates at low concentrations, and short lived areas where geochemically produced nucleotides can reside, would hinder evolution, particularly acting together. In contrast, a chaotic environment is not necessarily prohibitive, instead readily stimulating prebiotic evolution. That is, sporadic substrate availability (Yarus 2013, 2016) and a hazardous setting that allows only brief pool existence, putting pools in continuous danger (Fig. 5) ,and perhaps only allowing time for one cycle of selection (Fig. 5b; Table 1) are creative circumstances, potentially hosting the onset of biotic phenomena. In fact, young pools under strong selection are a uniquely creative class (Fig. 5b), commonly leaping under selection from infrequent templating to chemically useful, mostly encoded NppM (Eq. 10; Table 1).

And these changes can be relatively quick. Plots above span only months (e.g., Fig. 1), and the crucial transition can occur in a few days (Table 1). Thus, once two complementary, chemically activated nucleotides meet on a lifeless Hadean or Archean Earth, a recognizable genetic system can follow quite suddenly.

### Molecular Re-interpretation: The Template Catalysis Mechanism

Separation of these results into pool and molecular effects puts the findings in a new light. On one hand, pool accumulation of stable precursors enhances templating (Figs. 1a, 6b) and pool product selection moves synthesis toward templating (Fig. 5b; Table 1). On the other, the molecular character of the templating complex makes it uniquely selectable.

### Summarizing Synthesis in the Sporadically Fed Pool

**Scheme 2:**
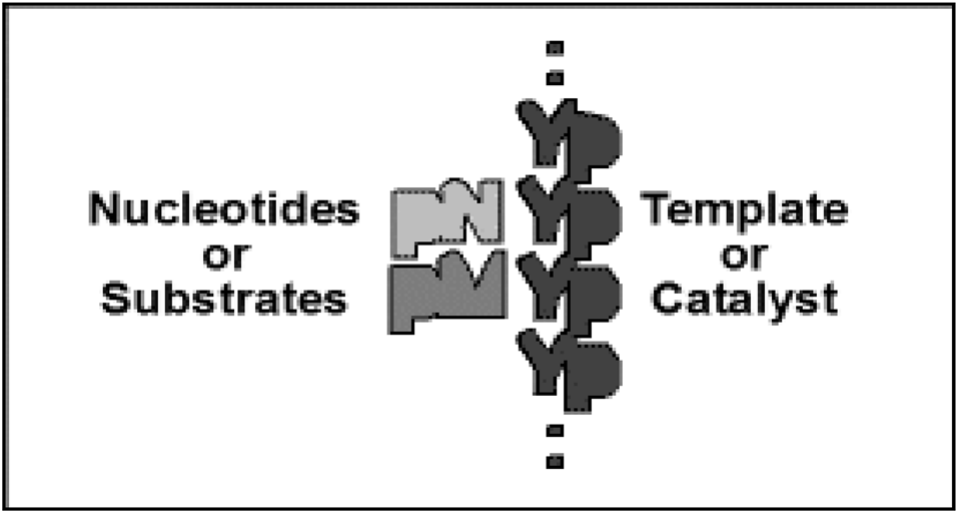
Generalized, low-resolution view of a reaction center. Ordering of nucleotide substrates on a template can be the same molecular event as assembly of an entropic catalytic center, which conjoins substrates for reaction

Synthesis of a potentially reactive 5′–5′ coenzyme congener is speeded by complementary templates (Puthenvedu et al. 2015; Majerfeld et al. 2016). Accordingly, templating, and so temp/chem, increases throughout a pool’s life (Fig. 1c; Eq. 9), making late pools very productive (Fig. 5a; Table 1). When templated product is useful, pools that emphasize elevation from templating will be selected (Fig. 6b), increasing yields (Fig. 6a). This accounts for more rapid progress in constant hazard, than in periodic hazard pools (Figs. 5b, 6a, b), and makes extended pool life very productive (Fig. 6b). So, except for pool effects, templates are selected (Scheme 2), particularly in young pools (Fig. 5b), because they bring dilute nucleotide substrates together. Modeling of helical complexes (Puthenvedu et al. 2015) shows that helical, base-paired 5′ nucleotides easily form a transition state for NppM synthesis, because adjacent 5′ nucleotide phosphates readily converge. What we normally term a template is innately also a type of catalyst (Scheme 2).

Thus RNA catalysis partially accounts for the rise of templating, but “catalysis” here differs from RNA world catalysis usually supposed. Thus for, e.g., the origin of translation, pure RNAs with complex higher-order structures can be isolated which perform all four essential reactions of protein biosynthesis, strongly arguing for translation’s comprehensive RNA origin (Yarus 2001). Here, instead of complex RNAs, the proposed first gene product requires only simple reactants undergoing simple adjacent bonding to a base-paired complement (Schemes 1, 2).

### Selection of Template Catalysis Requires No Specific Kinetic or Mathematical Precondition

Acting on template catalysis, quantitative selection favors qualitative change toward basic inherited behavior (Fig. 5b; Table 1; Scheme 2). This specifies the previously predicted route (Yarus 2016) by which structuring selection for a pre-genetic pool product would expedite appearance of a first inherited phenotype. The Scheme 2 mechanism was revealed by computation, but is plausible independent of any specific kinetic, chemical, or mathematical assumption. That is, selection of elevated template-catalyzed product is likely whenever product is useful. As an example: other nucleotides, differing significantly in structure and chemistry (compare (Pinheiro et al. 2012)), could plausibly take a similar pathway to a similar templated end. With generalized template selection in mind, it will seem extraordinary if similar events have not happened elsewhere, and many times.

## Methods

### Production of an Exponential Distribution of Lifetimes

Exponentially distributed lifetimes for a constant hazard pool (Fig. 3a) were generated by inverse transform sampling, meaning that the inverse of the cumulative exponential distribution function was evaluated using uniformly distributed pseudorandom numbers. Thus, lifetime = ln (*U*)/*k*_d_ (https://en.wikipedia.org/wiki/Inverse_transform_sampling) where *U* is a uniform random variate in the interval 0, 1 yields exponentially distributed lifetimes with the probability of failure *k*_d_ time^−1^.

### Behavior of a Realistic, Complete Cross-Templating System

**Scheme 3:**
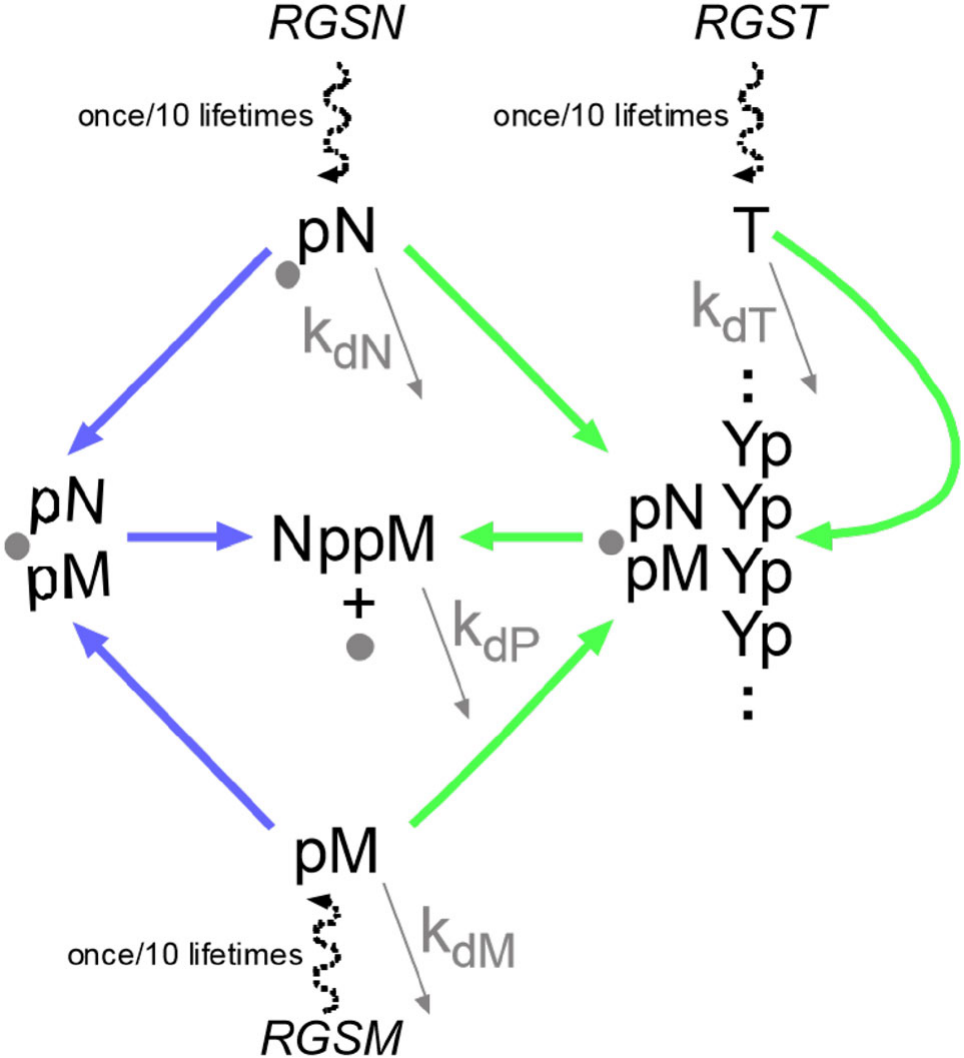
The NppM synthesis scheme modeled. The active product, a chemically reactive, cofactor-like 5′–5′ dimer NppM, appears via two routes; (1) termed chem; *blue arrows*, on the *left*: this is equivalent to second-order chemical reaction of stacked nucleotides via *k*_chem_ (M^−1^ lifetime^−1^). Also, (2) termed temp, *green arrows*, on the *right*: equivalent to base pairing of two nucleotides to template, then third-order reaction (Yarus, unpublished) with *k*_temp_ (M^−2^ lifetime^−1^). Time is measured in mean lifetimes of the least stable reactant, activated nucleotide (about 20 h at 12 °C) but can be read as “days,” for simplicity. The *small gray circle* is an activating group, 2-me-imidazole here (Joyce et al. 1984). Though pN is shown as activated, either nucleotide can be the activated one. Nucleotide supplies are variable, arriving at uncorrelated, random times (RGSM, RGSN = Random time, Gaussian Supplies of M & N) alongside Random time, Gaussian Supplies of Template (RGST), and in varied amounts >0 (Gaussian mean usually 1.22 mM here, ±0.61 mM (SD)). Nucleotides arrive randomly, averaging 1 arrival/10 lifetimes. Nucleotide N and M, template T, and product P decay at individual first-order rates *k*_dN_ and *k*_dM_, *k*_dT_ and *k*_dP_ lifetime^−1^, respectively (utilized in Supplementary Information)

Pools were simulated by casting Scheme 3 as differential equations (see Supplementary Information), and integrating the resulting system with the Rosenbrock integrator (Rosenbrock 1963) of Berkeley Madonna v.8.3.23.0 (simulation code is in Supplementary Information). Subsequent numerical analysis of kinetic results was performed in Microsoft Excel 2013. For example, constant hazard pools were investigated by calculation of 1000 time courses in Berkeley Madonna. Then, exponentially distributed lifetimes (Fig. 3a) were generated in Excel. Spreadsheet lookup functions were used to find corresponding exponentially distributed times and product concentrations among tabulated, pre-generated time courses.

### Data for Pool Calculations

#### Source of the Constants

Kinetic constants (12 °C), synthesis rates and standard errors come from the six pG/2MeImpG ratio experiment published previously (Fig. 4 of Majerfeld et al. 2016), fitted using a kinetic model like Scheme 3, which also accounts for decay of all reactants, the product and the template, as well as for the elevation of pG when 2MeImpG hydrolyzes.

Minimal stabilities were chosen (implying maximal decays) when literature values were interpreted, in order to avoid overestimation of pool output. Nucleobases are very stable; the least stable being C (deaminates to *U*) with a half-life of 2.5 × 10^4^ years (Levy and Miller 1998). The glycosidic bond of deoxyadenosine at modest temperature has a lifetime of 180 years (Wolfenden 2014). Ribonucleotides are instead likely to be limited by ribose (Larralde et al. 1995) with an aqueous half-life of 63 years. A 50 year lifetime implies decay at 4.6 × 10^−5^ per imidazolide lifetime, the rate used for nucleotides here. Ribophosphodiester bonds at 0 °C can have lifetimes of the order of 100 years (Soukup and Breaker 1999), so a 100-mer would suffer about 1 hydrolysis/year. A year is 4.38 × 10^2^ imidazolide lifetimes, so RNA decay is parsimoniously estimated as (4.38 × 10^2^)^−1^ = 2.3 × 10^−3^ lifetime^−1^. System product, GppG, showed no detectable decay in 20 days under our conditions. If “detectable” is estimated as <95% survival, then GppG decay is <2.1 × 10^−3^ lifetime^−1^, which is the stability used in calculations.

In Table 2, MeImpG appears less stable in reactions than it is when stored in buffered solutions, perhaps because of hydrolysis catalyzed by other nucleotides (Kanavarioti et al. 1992). Average (mean) molecular lifetimes are around 20 h (half-life = *t*_½_ = ln 2 * mean lifetime ≈ 17 h). Here, 2MeImpG appears insignificantly more unstable in the presence of poly(C), and so is assigned the same lifetime in all reactions (compare Puthenvedu et al. 2015).

**Table 2:** Modeling rates and stabilities—kinetic and decay constants used

Name		*t* = hours	*t* = lifetimes	Comment
Templated, green	*k*_temp_	82 ± 6 M^−2 h−1^	1640 M^−2^ life^−1^	val ± sem (Majerfeld et al. 2016); 3rd order
Chemical, blue	*k*_chem_	0.1 ± 0.02 M^−1 h−1^	2 M^−1^ life^−1^	As above, table; 2nd order
2meImpN → pN	*k*_dN_	0.041 ± 0.018 h^−1^	1 life^−1^	Above; no polymer; set = 0.05 h^−1^; 1st order
2meImpN → pN	*k*_dN_	0.055 ± 0.007 h^−1^	1 life^−1^	Above; + polymer; set = 0.05 h^−1^; 1st
pN decay	*k*_dM_	2.2 × 10^−6 h−1^	4.6 × 10^−5^ life^−1^	Assumes 50 year life (legend); 1st order
NppN decay	*k*_dP_	<1 × 10^−4 h−1^	2.1 × 10^−3^ life^−1^	Max 1st order; (Majerfeld and Yarus, unpub)
100-nt RNA	*k*_dT_	1.1 × 10^−4 h−1^	2.3 × 10^−3^ life^−1^	Literature value for 100-mer; 1st order

## Electronic supplementary material

Below is the link to the electronic supplementary material.

Supplementary material 1 (PDF 65 kb)

## Footnotes

1 Total templated synthesis = temp = ∫_0_^lifetime^ *k*_temp_(*N*)(*M*)(template)d lifetime. See “Methods” for symbols.

2 Total un templated synthesis = chem = ∫_0_^lifetime^ *k*_chem_(*N*)(*M*)d lifetime. See “Methods” for symbols.
