# Supplementary material for: Efficient Heritable Gene Expression Readily Evolves in RNA Pools
Source: J Mol Evol. 2017 Jul 1;84(5):236–52. doi: 10.1007/s00239-017-9800-1 (PMC5501911; doi:10.1007/s00239-017-9800-1)
Supplement: Supplementary file 1 — Supplementary material 1 (PDF 65 kb) [file 239_2017_9800_MOESM1_ESM.pdf]

```
METHOD stiff                                       ; variable-step RK5 (auto) or Rosenbrock (stiff)
           {monty2h,mmd}
           {Random-time, Gaussian spikes, then cross-templated ribodimer synthesis, template
synthesis, and decay; MY, 1/2016}
           {new spike mechanism for three substrates, and capable of cross-templated scheme}
           {includes spikes of 1 & 2 (activated nt), decay of 2 -> 1{can be commented out}, spikes of
3 (template), synthesis of 1_2}
           {incorporates low-concentration form of rate equation, with 2nd and 3rd order rates for
free and polymer-bound synthesis; uses expt'l rates from poly (C) ms}

; display chem, temp, conc1, conc2, conc3, conc1_2

STARTTIME = 0                                      ; housekeeping entries
STOPTIME = 1000
DTmin = 1e-10
DTmax = 1e-3                                       ; useful to control stiff integration
Tolerance = 1e-8                             ; low!
DTout = 0.5                                        ; partial output slightly faster


           {===rate & other system constants===}
decayconst1 = 4.6e-5                               ; rate of decay of pN in *lifetime^-1* units
decayconst2 = 1                                    ; decay of activated pN or pR, lifetime^-1; could
be due to activating group
decayconst3 = 2.3e-3                               ; decay of template polymer, presumably hydrolytic
decayconst1_2 = 2.1e-3                             ; lifetime^-1, RppA, could have unstable reactive
group
rt = 1640                                    ; /M^2 /life, 82 /hr x 20 hr; 3rd order reaction at
templated nt interface; from poly(C) ms, Table 1
rc = 2                                             ; /M /life, 0.1 /hr x 20 hr; 2nd order reaction at
solution stack interface; from poly (C) ms, Table 1
SpikeInt = 10                                      ; mean interval between spikes in life units

           {===spikes of reactant 1, NMP===}
totspikes1 = stoptime/SpikeInt                           ; # spikes of react 1 in all, start ->
stoptime
threshold1 = totspikes1*stepsize/stoptime                ; spike threshold for totspikes using 0 -> 1
interval; probably 1st cycle -> 0
```

```
spikesize1 = 0.001                                  ; mean spike size, molar
spikeSD1 = 0.0005                             ; SD of spike size

            {===spikes of reactant 2, activated nt===}
totspikes2 = stoptime/SpikeInt                            ; # spikes of react 2 in all, start ->
stoptime
threshold2 = totspikes2*stepsize/stoptime                 ; spike threshold for totspikes using 0 -> 1
interval
spikesize2 = 0.001                                  ; mean spike size, molar
spikeSD2 = 0.0005                             ; SD of spike size

            {===spikes of reactant 3, polymer===}
totspikes3 = stoptime/SpikeInt                            ; # spikes of react 3 in all, start ->
stoptime
threshold3 = totspikes3*stepsize/stoptime                 ; spike threshold for totspikes using 0 -> 1
interval
spikesize3 = 1e-3                             ; mean spike size, molar nt phpsphate
spikeSD3 = 5e-4                               ; SD of spike size

            {===init condx===}
init chem = 1e-25
init temp = 1e-30

init conc1 = 1e-29
init conc1_2 = 1e-28
init spiketime1 = 0
init spikeslope1 = 0
init gaussspikedist1 = normal (spikesize1, spikeSD1)
init ssz1 = DTmin

init conc2 = 1e-27
init spiketime2 = 0
init spikeslope2 = 0
init gaussspikedist2 = normal (spikesize2, spikeSD2)
init ssz2 = DTmin

init conc3 = 1e-26
init spiketime3 = 0
```

```
init spikeslope3 = 0
init gaussspikedist3 = normal (spikesize3, spikeSD3)
init ssz3 = DTmin


           {===spike generator 1===}
next spiketime1 = if random (0,1) < threshold1 then 1                     ; reset spiketime to give a
spike
      else
      if spiketime1 >= 1 AND spiketime1 <= 10 then spiketime1 + 1         ; internal spike count
advances
           else
           0                                             ; spiketime counts 1 -> 11, returns to 0


next ssz1 = if spiketime1 >= 1 then ssz1                                  ; have spike conserve step
size
      else
      stepsize                                           ; ssz alters after spike

limit gaussspikedist1 >= 0                                     ; spike size >= 0
next gaussspikedist1 = if spiketime1 >= 1 then gaussspikedist1                  ; conserve spike size
during spike
           else
           normal (spikesize1, spikeSD1)                             ; the Madonna way to normal
variation; note BM error in "normal (mean, SD)": SD, not Var!

next spikeslope1 = if spiketime1 >= 1 then gaussspikedist1/(10*ssz1)
           else
           0                                             ; gaussian spike, converted to slope, and
held const.during 11 spiketime intervals


           {===spike generator 2===}
next spiketime2 = if random (0,1) < threshold2 then 1                     ; reset spiketime to give a
spike
      else
```

```
      if spiketime2 >= 1 AND spiketime2 <= 10 then spiketime2 + 1         ; internal spike count
advances
            else
            0                                             ; spiketime counts 1 -> 11, returns to 0


next ssz2 = if spiketime2 >= 1 then ssz2                                  ; have spike conserve step
size
      else
      stepsize                                            ; ssz alters after spike

limit gaussspikedist2 >= 0                                      ; spike size >= 0
next gaussspikedist2 = if spiketime2 >= 1 then gaussspikedist2                  ; conserve spike size
during spike
            else
            normal (spikesize2, spikeSD2)                               ; the Madonna way to normal
variation; note BM error in "normal (mean, SD)": SD, not Var!

next spikeslope2 = if spiketime2 >= 1 then gaussspikedist2/(10*ssz2)
            else
            0                                             ; gaussian spike, converted to slope, and
held const.during 11 spiketime intervals


            {===spike generator 3===}
next spiketime3 = if random (0,1) < threshold3 then 1                     ; reset spiketime to give a
spike
      else
      if spiketime3 >= 1 AND spiketime3 <= 10 then spiketime3 + 1         ; internal spike count
advances
            else
            0                                             ; spiketime counts 1 -> 11, returns to 0


next ssz3 = if spiketime3 >= 1 then ssz3                                  ; have spike conserve step
size
      else
      stepsize                                            ; ssz alters after spike
```

```
limit gaussspikedist3 >= 0                                    ; spike size >= 0
next gaussspikedist3 = if spiketime3 >= 1 then gaussspikedist3                 ; conserve spike size
during spike
           else
           normal (spikesize3, spikeSD3)                           ; the Madonna way to normal
variation; note BM error in "normal (mean, SD)": SD, not Var!

next spikeslope3 = if spiketime3 >= 1 then gaussspikedist3/(10*ssz3)
           else
           0                                             ; gaussian spike, converted to slope, and
held const.during 10 spiketime intervals

No = conc1 + conc2                                            ; summed nt, for stacking calculations
involving No = total nt
tot = chem + temp                                       ; total dimer synthesis, withhout other
effects
ratio = chem/tot                                              ; fraction of total synthesis that is
chemical

           {===integrate reactant 1 +/- spike===}
d/dt (conc1) =  spikeslope1 {+decayconst2*conc2} -decayconst1*conc1 -conc1*conc2*(rc + (conc3*rt))

           ; reactant in 10 equal steps, with decay, 2 -> 1 in the mix - comment out easy
           {===integrate reactant 2 +/- spike===}
d/dt (conc2) =  spikeslope2 -decayconst2*conc2  -conc1*conc2*(rc + (conc3*rt))                   ;

      ; reactant addn in 10 equal steps, with concurrent decay
           {===integrate spikes of reactant 3===}
d/dt (conc3) = spikeslope3 -decayconst3*conc3                            ; no consumption of polymer,
component #3

           {===integrate 1_2 synthesis & decay, eg, RppA===}
d/dt (conc1_2) = conc1*conc2*(rc + (conc3*rt)) -decayconst1_2*conc1_2
                                                        ; 1st term, chem stacked, 2nd, templated
stacked
```

```
d/dt (chem) =  conc1*conc2*rc                                       ; total synthesis from chemical
stacks

d/dt (temp) =   conc1*conc2*conc3*rt                                ; total synthesis from templated
stacks
```
